# Supplementary material for: Exploring Knowledge of Antibiotic Use, Resistance, and Stewardship Programs among Pharmacy Technicians Serving in Ambulatory Care Settings in Pakistan and the Implications
Source: Antibiotics (Basel). 2022 Jul 8;11(7):921. doi: 10.3390/antibiotics11070921 (PMC9311796; doi:10.3390/antibiotics11070921)
Supplement: Supplementary file 1 [file antibiotics-11-00921-s001.zip › antibiotics-1793033-supplementary.pdf]

Supplementary Material - Questionnaire

Section S1 **Demographic details:**

(Please select one option from the options given in front of each question)

- ✓ **Age (in years):** ______________________
- ✓ **Name of health facility:** ______________________
- ✓ **Gender:** A) Female B) Male
- ✓ **Marital Status:** A) Non-Married B) Married
- ✓ **Residence:** A) Rural B) Urban
- ✓ **Designation:** A) Junior pharmacy technician
  B) Senior pharmacy technician
- ✓ **Training/refresher course about medicines/antibiotics:** A) Yes B) No
- ✓ **Working Department:** A) Emergency pharmacy
  B) Indoor pharmacy
  C) Out door pharmacy
- ✓ **Years of Experiences:** A) 1-5 years B) 6-10 years C) 11-15 years
  D) 16-20 years E) More than 20 years
- ✓ **Number of prescriptions filled per day**
  A)1-30 B) 31-60 C) 61-100 D) 101-150 E) More than 151

Section S2: Understanding about antibiotic use

(Please select one option according to your understanding about antibiotic use).

Sr. No.	Variables	Yes	No	Don't know
1	Antibiotics are the most commonly prescribed anti-infective agents by public health-care sector facilities			
2	Common cold if treated with antibiotics will make the patients recover more quickly			
3	Antibiotics should be prescribed as preventive measures to fight against future microbial attacks			
4	Antibiotics cannot treat influenza			
5	Antibiotics are indicated to relieve pain			
6	Antibiotics might develop allergy in susceptible individuals			
7	Diphenhydramine is an antibiotic used in treating upper respiratory infections			
8	Cefotaxime belongs to the third-generation cephalosporins			
9	Patients can stop taking antibiotics when the symptoms are improving			

**10**	Keeping the left-over antibiotic course for the next time treatment of the same type of infection is a good practice			
**11**	Antibiotics treatment can eliminate most of the sensitive bacterial cells from patients			
**12**	Antibiotics can be obtained without a prescription in Pakistan			
**13**	Antibiotics are the first line of treatment in sore throat			

Section S3 : Understanding about antibiotic resistance

(Please select one option according to your understanding about antibiotic resistance)

**Sr. No.**	**Variables**	**Yes**	**No**	**Don't know**
**1**	A resistant bacterium cannot spread in healthcare institutions			
**2**	Health care workers serve as vectors carrying resistant strains from infected patients to normal patients			
**3**	Exposure to antibiotics appears to be the principal risk factor for the emergence of antibiotic resistant bacteria			
**4**	Inadequate duration of therapy contributes to antibiotic resistance leading to poor patient compliance			
**5**	Inadequate doses contribute to antibiotic resistance due to poorly designed dosing regimen			
**6**	Antimicrobial resistance can be minimized through changing empiric therapy to a selected narrow-spectrum therapy in response to the availability of culture and sensitivity results			
**7**	Cross-resistance is the condition in which resistance occurs to a particular antibiotic that often results in resistance to other antibiotics, usually from a similar class			
**8**	Lack of enforcement regulation sometimes permits antibiotics to be purchased without a prescription from pharmacies			

Section S4 Awareness about the terminologies related to antibiotic resistance

(Please select one option according to your awareness about terminologies related to antibiotic resistance)

Sr. No.	Variables	Yes	No
1	Did you hear about the term: 'Antimicrobial resistance'?		
2	Did you hear about the term: 'Antibiotic Resistance'?		
3	Did you hear about the term: 'Superbugs'?		
4	Did you hear about the term: 'Drug Resistance'?		
5	Did you hear about the term: 'Multidrug Resistance?		
6	Did you hear about the term: 'Extensively drug Resistance?		
7	Did you hear about the: use of antibiotics with caution?		
8	Did you hear about the: Antibiotic resistance spreading very fast?		
9	Did you hear about the: national action plan for antimicrobial resistance in Pakistan?		

Section S5 Awareness about the antimicrobial stewardship program (ASP)

(Please select one option according to your understanding about antimicrobial stewardship program (ASP))

Sr. No.	Variables	Yes	No	Don't know
1	Did you hear about the term Antimicrobial Stewardship Programs (ASPs)?			
2	Did you know the core components of Antimicrobial Stewardship Programs (ASPs)?			
3	Did you know antimicrobial stewardship programs promote reasonable prescription of antimicrobials			
4	Antimicrobial stewardship programs help control antimicrobial resistance			
5	Antimicrobial stewardship programs reduce the overuse of antimicrobials			
6	Antimicrobial stewardship programs reduce the cost of treatment.			
7	Antimicrobial stewardship programs Improves medical quality.			

- What is the most common source of information about antibiotic use, antibiotic resistance, terminologies related to antibiotic resistance and antimicrobial stewardship program?
  A. Books & journals
  B. Print & electronic media
  C. Social media like Facebook, WhatsApp etc.
  D. Health care professionals
  E. Leaflets/ broachers
  F. Online applications related to medicines
  G. Others.
